# Nitric oxide synthesis by nitrate reductase is regulated during development in *A* *spergillus*

**DOI:** 10.1111/mmi.13211

**Published:** 2015-10-14

**Authors:** Ana T. Marcos, María S. Ramos, Jose F. Marcos, Lourdes Carmona, Joseph Strauss, David Cánovas

**Affiliations:** ^1^ Departamento de GenéticaFacultad de BiologíaUniversidad de SevillaSevillaSpain; ^2^ Department of Food ScienceInstitute of Agrochemistry and Food Technology (IATA)ValenciaSpain; ^3^ Fungal Genetics and Genomics UnitDepartment of Applied Genetics and Cell BiologyUniversity of Natural Resources and Life Sciences (BOKU) ViennaViennaAustria; ^4^ Department of Health and EnvironmentBioresourcesAustrian Institute of Technology (AIT)ViennaAustria; ^5^ Present address: Deparment of Plant BiotechnologyFundo de Defesa da CitriculturaVila Melhado – AraraquaraSão PauloBrazil

## Abstract

Nitric oxide (NO) is a signalling molecule involved in many biological processes in bacteria, plants and mammals. However, little is known about the role and biosynthesis of NO in fungi. Here we show that NO production is increased at the early stages of the transition from vegetative growth to development in *A* *spergillus nidulans*. Full NO production requires a functional nitrate reductase (NR) gene (*nia* *D*) that is upregulated upon induction of conidiation, even under N‐repressing conditions in the presence of ammonium. At this stage, NO homeostasis is achieved by balancing biosynthesis (NR) and catabolism (flavohaemoglobins). *nia* *D* and flavohaemoglobin *fhb* *A* are transiently upregulated upon induction of conidiation, and both regulators AreA and NirA are necessary for this transcriptional response. The second flavohaemoglobin gene *fhb* *B* shows a different expression profile being moderately expressed during the early stages of the transition phase from vegetative growth to conidiation, but it is strongly induced 24 h later. NO levels influence the balance between conidiation and sexual reproduction because artificial strong elevation of NO levels reduced conidiation and induced the formation of cleistothecia. The nitrate‐independent and nitrogen metabolite repression‐insensitive transcriptional upregulation of *nia* *D* during conidiation suggests a novel role for NR in linking metabolism and development.

## Introduction

Reproduction is an essential part of the biological cycle of fungi and a mode of dispersion in the environment. Among fungi, *Aspergillus nidulans* has been used as a model organism to study reproduction for decades. *A. nidulans* displays two modes of reproduction: sexual or asexual. The asexual development program (conidiation) is initiated when superficial hyphae are exposed to an air interphase. The asexual reproductive structure of *A. nidulans* is the conidiophore, which forms green‐pigmented conidiospores approximately 24 h after induction of development (Adams *et al*., 1998; Etxebeste *et al*., 2010; Yu, 2010). A central regulatory pathway encompassing BrlA, AbaA and WetA controls conidiation (see reviews by Adams *et al*., 1998; Etxebeste *et al*., 2010; Yu, 2010, and references therein). The first component in the regulatory cascade, BrlA, is essential to drive conidiation (Adams *et al*., 1988). *brlA* is not expressed during vegetative growth, but its induction during development is controlled by a number of genes, e.g. the *fluffy* genes (Adams *et al*., 1998; Yu *et al*., 2006). These *fluffy* genes are expressed in vegetative mycelium and are able to respond to stimuli to induce the co‐ordinated activation of the master regulator *brlA* (Etxebeste *et al*., 2010).

The sexual program is promoted by signals that can be grouped in four categories: absence of light, nutrient availability, temperature and atmospheric gases. Under laboratory conditions, plate sealing limits the exchange of gases and results in lowering the oxygen levels and increasing the CO_2_ levels, which promotes sexual reproduction (Dyer and O'Gorman, 2012). *A. nidulans* is homothallic, i.e. each strain harbours both mating type genes, *MAT1/matB* and *MAT2/matA* (Paoletti *et al*., 2007). During this program, *A. nidulans* develops spherical fruiting bodies called cleistothecia, which contain meiospores called ascospores (Dyer and O'Gorman, 2012). Genes involved in the regulation of cleistothecia formation were mainly identified through *A. nidulans* mutants defective at distinct stages of sexual development: acleistothecial strains such as Δ*nsdD* and Δ*stuA* (Wu and Miller, 1997; Han *et al*., 2001), mutations that stop the propagation program after production of Hülle cells such as Δ*steA* and Δ*medA* (Clutterbuck, 1969; Vallim *et al*., 2000) and strains that are blocked in the maturation of cleistothecia (Eckert *et al*., 2000; Hoffmann *et al*., 2000; Busch *et al*., 2001).

Early work in H. Ninnemann's laboratory connected nitrate reductase activity to blue light‐promoted conidiation in *Neurospora crassa* (Klemm and Ninnemann, 1979; Ninnemann, 1991). The pathway for the assimilation of nitrate has been extensively studied in *A. nidulans* for several decades. Nitrate assimilation requires the action of two enzymes: nitrate reductase (NR, encoded by *niaD*), mediating the reduction of nitrate to nitrite; and the nitrite reductase (NiR, encoded by *niiA*) involved in the conversion of nitrite to ammonium. Nitrate assimilation is generally repressed in fungi by the presence of the preferred nitrogen sources ammonium or glutamine and, in *A. nidulans*, is activated by nitrate or nitrite through the synergistic action of the pathway specific regulator NirA and the general regulator of nitrogen metabolism AreA (Arst and Cove, 1973; Caddick *et al*., 1986; Burger *et al*., 1991; Marzluf, 1997; Strauss *et al*., 1998; Muro‐Pastor *et al*., 1999; Todd *et al*., 2005; Berger *et al*., 2006; 2008; Bernreiter *et al*., 2007; Schinko *et al*., 2010).

Recently, the regulation of nitrate assimilation was connected to nitric oxide (NO) metabolism in *A. nidulans* (Schinko *et al*., 2010). NO is widely recognised for its role as signalling compound from bacteria to mammals (Rosselli *et al*., 1998; He *et al*., 2004; Kwon *et al*., 2012; Seth *et al*., 2012). This diatomic molecule is a short‐lived radical with a half‐live of a few seconds. It plays essential regulatory roles in a variety of biological processes but can also cause nitrosative stress to cells. Flavohaemoglobins are known to function in NO detoxification, converting the reactive NO radical into nitrate and consequently protecting against nitrosative stress derived from either exogenous or endogenous sources (Gardner *et al*., 1998a; Foster *et al*., 2009). Different routes for cellular NO biosynthesis have been described in nature, including both oxidative and reductive pathways. The oxidative synthesis has been reported in bacteria, plants and mammals involving the conversion of L‐arginine and O_2_ into citrulline and NO by the nitric oxide synthase (Alderton *et al*., 2001; Lamotte *et al*., 2005; Gorren and Mayer, 2007). The reductive NO synthesis was reported as first in legumes (Dean and Harper, 1988; Yamasaki, 2000), but later was shown to be widespread in plants, and it involves the action of the nitrate reductase at saturating nitrite concentrations under reductive conditions (excess of NAPDH) (Yamasaki, 2000; Yamasaki and Sakihama, 2000; Rockel *et al*., 2002; Lamotte *et al*., 2005). In mammals alternative reductive pathways for the conversion of nitrite to NO have been identified involving different enzymes such as cytochrome *c*, P450 or deoxyhaemoglobin in the heart and wall vessels or by non‐enzymatic mechanisms (Zweier *et al*., 2010). Notably, fungal genomes are devoid of NO synthase homologues (Gorren and Mayer, 2007; Samalova *et al*., 2013) and the physiological and genetic mechanisms that determine changes in the level of NO in fungi are poorly understood in these organisms. Addition of NO chemical donors induces asexual spore development in the ascomycete *N. crassa* (Ninnemann and Maier, 1996), sporangiophore development in the zygomycete *Phycomyces blakesleeanus* (Maier *et al*., 2001), conidiation in *Coniothyrium minitans* (Gong *et al*., 2007) and sexual fruiting bodies in the ascomycete *A. nidulans* (Baidya *et al*., 2011) and in the basidiomycete *Flammulina velutipes* (Song *et al*., 2000). *Magnaporthe oryzae* synthesised NO during germination and early development; however, the deletion of candidate genes in both oxidative and reductive routes did not impair NO biosynthesis in this fungus (Samalova *et al*., 2013). In *A. nidulans* the two flavohaemoglobins FhbA and FhbB were shown to be involved in the metabolism of NO to nitrate (Gardner *et al*., 1998b; Poole and Hughes, 2000). In addition, they are essential to protect nitrate reductase and nitrite reductase enzymes against nitrosative damage elicited by growth on nitrite at low environmental pH conditions (Schinko *et al*., 2010; 2013).

In this work, we provide data indicating the existence of a nitrate reductase‐dependent pathway for the synthesis of NO in *A. nidulans* and found that NO levels increase immediately after switching from vegetative growth to conidiation. Both flavohaemoglobins are involved in the balance of NO during the developmental switch.

## Results

### Aspergillus produces NO

**Figure 1:**
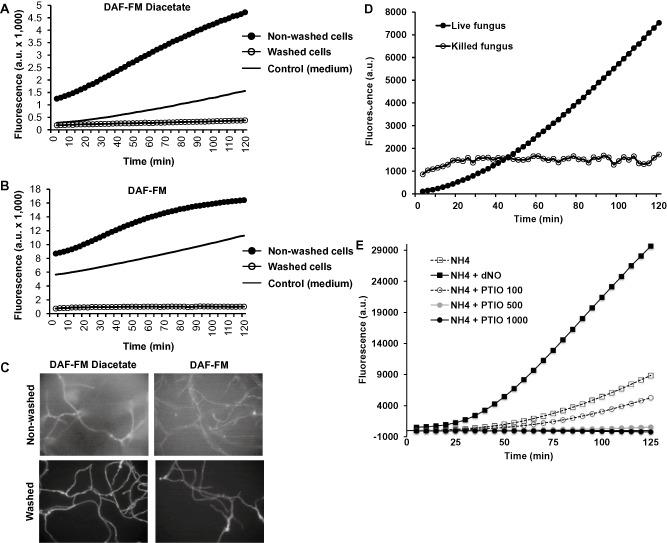
Quantification of NO produced by *A* *spergillus*. *A* *. nidulans* was grown in liquid ammonium minimal medium. DAF‐FM DA (A) or DAF‐FM (B) was added to the cultures and incubated for 20 min in the dark. After cell loading of the dyes, one sample containing each dye was washed three times, whereas control samples were not washed. Fresh medium was used as an additional control to detect the background signal. Fluorescence was monitored by fluorometry (A and B) or cells were imaged by fluorescent microscopy (C). (D) *A* *. nidulans* was grown in liquid ammonium minimal medium for 16 h. One sample was kept as a living control, whereas the rest of the cells were killed by autoclaving. DAF‐FM DA was added to the samples and the fluorescence was recorded by fluorometry. Background signal obtained with fresh medium was subtracted from signals obtained with both cell samples. (E) *A* *spergillus* cells were grown in the presence or in the absence of the NO‐releasing compound dNO (1.5 mM) or different concentrations of the NO‐scavenger PTIO (100–1000 μM). DAF‐FM DA was added to the samples and fluorescence was recorded by fluorometry. Under all these conditions, fungal growth was similar. In all cases, one representative experiment is shown.

Several techniques have been employed to detect and quantify NO production (Yamasaki and Sakihama, 2000; Maier *et al*., 2001; Planchet and Kaiser, 2006; Nagano, 2009; Samalova *et al*., 2013). We selected the NO‐sensitive fluorescent probes developed by Nagano and collaborators (Kojima *et al*., 1998a, 1998b, 1999). This approach was previously reported to be useful in fungi (Carmona *et al*., 2012; Samalova *et al*., 2013). DAF‐FM is very sensitive to NO, with a detection limit of 3 nM, whereas DAF‐FM diacetate (DAF‐FM DA) is cell‐permeable and passively diffuses across cellular membranes. Once inside the cells it is deacetylated to DAF‐FM by esterases (Kojima *et al*., 1999). To test whether they could also be employed in *Aspergillus*, fungal cells were grown in liquid ammonium minimal medium. Either DAF‐FM or DAF‐FM DA was added to the cultures. After cells were loaded with the dyes for 20 min, the excess of both dyes was washed out in half of the samples, and all of them were transferred to a 96‐well plate for monitoring fluorescence. As shown in Fig. 1A–B, there was an increase in the fluorescence over time when the excess of dye were not washed off the cells. However, when the cells were washed, the fluorescence recorded was drastically lower than in non‐washed cells or in control medium. To analyse whether the monitored fluorescence was the result of the reaction with the NO‐sensitive probe inside or outside the cells, fluorescence was imaged under a fluorescent microscope (Fig. 1C). When the cells were not washed, fluorescence was observed both outside and inside the cells regardless of the dye (DAF‐FM or DAF‐FM DA). After the excess of dye was washed out, fluorescence was observed preferentially inside the cells. To confirm the biological origin of the signal, *Aspergillus* cells grown for 16 h in liquid ammonium minimal medium were autoclaved. DAF‐FM DA was added to live cells, dead cells and the minimal medium and fluorescence was recorded (Fig. 1D). While fluorescence in live cell samples increased continuously with time, the fluorescence generated in the samples containing heat‐killed fungi remained constant over time, suggesting that NO production was mediated by live cells.

It has been previously reported that the DAF family of NO‐sensitive dyes can also react with ascorbic acid and dehydroascorbic acid (Zhang *et al*., 2002a, 2002b). Although this possibility was already disregarded by Samalova and collaborators in *Magnaporthe* (Samalova *et al*., 2013), we tested this possibility in *Aspergillus* by employing the NO‐scavenger 4,4,5,5‐tetramethylimidazoline‐l‐oxyl3‐oxide (PTIO). Addition of different concentrations of PTIO resulted in a dose‐dependent decrease of fluorescence compared with the control sample in the absence of the NO scavenger (Fig. 1E). Addition of the NO‐releasing compound detaNONOATE (dNO) produced a high increase of fluorescence, as expected. Collectively, it shows that DAF‐FM and DAF‐FM DA were able to detect the NO produced by *Aspergillus*, both intracellular and extracellularly. We selected DAF‐FM DA for the rest of the study for having a better signal ratio between samples containing cells and control media in our 96‐well plate assays (3.3 with DAF‐FM DA versus 1.5‐fold with DAF‐FM).

### NO synthesis during growth in liquid media partially depends on niaD

**Figure 2:**
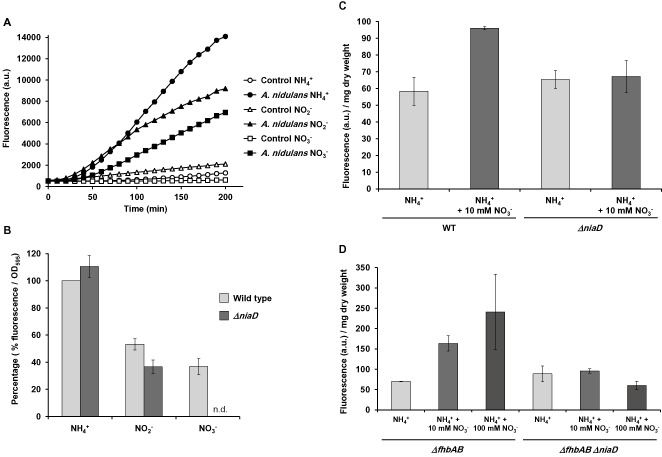
NO quantification in mycelium in liquid medium. A. *A* *. nidulans* wild type was inoculated in media containing the indicated compounds as sole nitrogen sources. DAF‐FM DA was added to the cultures and non‐inoculated media (control samples). Media, which was not inoculated, was employed as control to determine the biological origin of the NO produced. Fluorescence was recorded over time. One representative experiment is shown. B. *A* *. nidulans* wild type (light grey bars) and *Δnia* *D* (dark grey bars) strains were grown in liquid media with the indicated nitrogen sources. The *Δnia* *D* strain could not grow in nitrate, and consequently, NO production was not tested in this sample (n.d.). Data shown correspond to one time point after addition of DAF‐FM DA (100 min) and are referred to the wild‐type strain grown in ammonium media (100%). Data shown are the mean and the standard error of the mean of at least three independent biological replicates. N.d.: not determined. C. *A* *. nidulans* wild type and *Δnia* *D* strains were grown in ammonium liquid medium for 12 h. The mycelia were filtered, washed and transferred to ammonium (control) and ammonium + 10 mM nitrate. The cultures were grown for 6 additional hours. DAF‐FM DA was added to quantify NO production, and fluorescence was recorded for 60 min. Data shown are the mean and the standard error of the mean of two independent biological replicates. D. *A. nidulans Δfhb* *A* *Δfhb* *B* and *Δfhb* *A* *Δfhb* *B* *Δnia* *D* strains were grown in ammonium liquid medium for 12 h. The mycelia were filtered, washed and transferred to ammonium (control), ammonium + 10 mM nitrate, and ammonium + 100 mM nitrate. The cultures were grown for 6 additional hours. DAF‐FM DA was added to quantify NO production, and fluorescence was recorded for 60 min. Data shown are the mean and the standard error of the mean of two independent biological replicates.

It has been known for a long time that fungi can produce NO (Ninnemann and Maier, 1996); however, the mechanisms of NO synthesis have remained obscure so far. In order to study a possible role of the nitrate assimilation pathway in the biosynthesis of NO, our experimental set up was as follows: the *A. nidulans* wild type strain was grown in liquid media containing nitrate, nitrite or ammonium as sole nitrogen source for 16 h; then NO levels were followed for up to 200 min by addition of the NO‐sensitive fluorescent dye DAF‐FM DA directly to the growth medium and by subsequent fluorometric quantification (Fig. 2A). The level of fluorescence intensity increased in the samples containing fungal cells over time, to a higher extent than in control samples (which contain only the culture media). Subtraction of the background signal derived from the growth media confirmed that, interestingly, NO levels were higher in ammonium than in nitrite or nitrate‐containing media (Fig. 2B, wild type strain).

We then investigated whether a nitrate reductase‐dependent route for the synthesis of NO is operational in *A. nidulans*, similar to the situation described in plants (Dean and Harper, 1988; Yamasaki and Sakihama, 2000; Rockel *et al*., 2002). We employed a *ΔniaD* mutant lacking nitrate reductase. Obviously this mutant cannot grow on nitrate as sole nitrogen source, but it can grow on nitrite, which is, in plants, the substrate for the nitrate reductase to produce NO. No major differences were found in the NO levels between the wild type and the mutant strain on ammonium (Fig. 2B). However, when both strains were grown in nitrite, the *ΔniaD* mutant produced 30% less NO than the wild type, indicating that this enzyme participates in the production of NO and is responsible for around 30% of this metabolite synthesis when nitrite served as N‐source. On nitrate medium, the wild type strain showed the smallest amount of NO compared with the other two types of nitrogen (Fig. 2B). In order to test also NO production of *ΔniaD* on nitrate, the strains were pre‐grown on ammonium for 12 h and then transferred to nitrate‐containing ammonium liquid medium or back to sole ammonium medium as control. After 6 h of growth under these conditions, DAF‐FM DA was added to the cultures, and fluorescence was quantified (Fig. 2C). The wild‐type strain showed higher NO levels than the mutant when the medium was supplemented with nitrate indicating again that NR is involved in the production of NO through the nitrate assimilation route.

Our data suggested a putative role for *niaD* in mediating the synthesis of NO. Previous data reasoned that the flavohaemoglobin genes could be involved in the catabolism and thus detoxification of NO (Schinko *et al*., 2010). The deletion of both flavohaemoglobin genes, *fhbA* and *fhbB*, did not affect NO levels in ammonium medium (Fig. 2C–D, compare NO levels with the wild type strain), but there was a drastic increase in NO levels much higher than in the wild type (up to 3.5‐fold increase) when nitrate was present in the medium (Fig. 2D). Importantly, even in the presence of the repressing nitrogen source ammonium, nitrate was an obvious source for NO production. This is in agreement with findings by Schinko *et al*. (2010), who showed that the transcription of *fhbA* is independent from AreA function and that full induction of this gene also occurs under induced‐repressed conditions (i.e. ammonium plus nitrate). When the nitrate reductase *niaD* was deleted in the flavohaemoglobin deletion mutant (*ΔniaD ΔfhbA ΔfhbB*), the increase of NO in the presence of nitrate was not observed, confirming that a nitrate pathway operates to synthesise NO in *A. nidulans* and that this pathway requires a functional *niaD* gene.

### Deletion of niaD results in drastic reduction of NO production during growth on solid media

**Figure 3:**
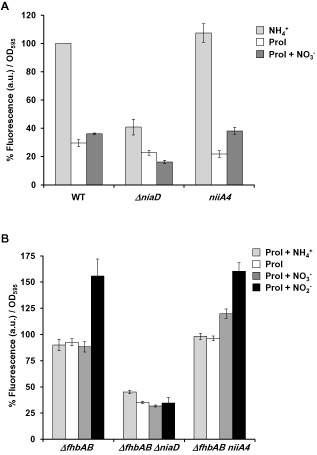
NO quantification on solid medium. A. *A* *. nidulans* wild‐type, *Δnia* *D* and *nii* *A* *4* strains were grown on solid media containing the indicated nitrogen sources at 10 mM (ammonium or nitrate) or 3 mM (proline) at 37°C for 18 h. DAF‐FM DA was added to quantify NO production, and fluorescence was recorded for 100 min. Data shown are referred to the wild‐type strain grown on ammonium media (100%). Data shown are the mean and the standard error of the mean of at least three independent biological replicates. B. *A* *. nidulans Δfhb* *A* *Δfhb* *B* *, Δfhb* *A* *Δfhb* *B* *Δnia* *D* and *Δfhb* *A* *Δfhb* *B* *nii* *A* *4* strains were grown on proline solid media for 18 h. DAF‐FM DA was added to quantify NO production. Then, the indicated nitrogen sources were added, and fluorescence was recorded for 60 min. Data shown are referred to the control strain *(Δfhb* *A* *Δfhb* *B*) grown on proline without the addition of any other nitrogen source. Error bars represent the standard error of the mean.

Previous transcriptomic data obtained in our laboratory suggested that the nitrate utilisation pathway was expressed during the induction of conidiation (Canovas *et al*., 2014), which allowed us to hypothesise that, in addition to vegetative growth, NO could also be produced through the nitrate pathway during development. For this reason, we were interested in investigating whether NR was also relevant for NO production on solid medium. We compared the NO levels in the wild type and the *ΔniaD* strain. Both strains were grown on solid medium containing different nitrogen sources (ammonium, proline or proline supplemented with nitrate). Proline allows the growth of the wild type and also the *ΔniaD* strain, and it is a neutral (non‐repressing, non‐inducing) nitrogen source for the nitrate assimilation system. Surprisingly, we found that NO levels of the *ΔniaD* mutant on ammonium solid medium was only 41% of the wild type (Fig. 3A), whereas in liquid ammonium medium both exhibited similar levels (Fig. 2). On proline solid medium, the *ΔniaD* mutant produced less NO compared with the wild type. When nitrate was added to the proline solid media, the wild type increased NO production (*P* < 0.05; Student's *t*‐test), whereas in the *ΔniaD* strain reduced levels were found: 30% less relative to proline media (*P* < 0.05; Student's *t*‐test), and 55% less NO than the wild type in proline plus nitrate (*P* < 0.01; Student's *t*‐test). In this case, we observed that the NO levels in the nitrite reductase defective mutant (*niiA4*) were similar to the wild‐type levels (Fig. 3A). Therefore, all these data suggest that there is a *niaD*‐dependent pathway for the synthesis of NO both in liquid and on solid media and that additional pathway(s) for the synthesis of NO must operate in *A. nidulans*.

With the aim to confirm the source of NO, the flavohaemoglobin mutants were used again to reduce NO catabolism and thus to increase the overall levels of this metabolite. The three strains were grown on solid media containing proline as a sole nitrogen source; the fluorescence recording was started by addition of DAF‐FM, and then different nitrogen sources were individually added to the assay as the recording continued. As shown in Fig. 3B, addition of nitrate did not have major effects on *ΔfhbA ΔfhbB*. However, addition of nitrite elicited an increase in the fluorescence. The nitrite reductase mutant (*ΔfhbA ΔfhbB niiA4*), on the other hand, exhibited an increase in the NO levels after the addition of either nitrate or nitrite, but not ammonium, demonstrating that the nitrite reductase (NiR) was not responsible for the synthesis of NO. Addition of either nitrate or nitrite to the *ΔfhbA ΔfhbB ΔniaD* mutant did not increase the NO levels over the control sample; furthermore, this strain showed reduced NO levels in the control conditions (proline) compared with the wild type (*ΔfhbA ΔfhbB)* and the *ΔfhbA ΔfhbB niiA4* strains (Fig. 3B). Addition of ammonium to any of the strains did not change the NO levels significantly over the control condition (proline), as expected. Therefore, taken together, our data demonstrate that the nitrate reductase is partially responsible for the synthesis of NO employing nitrite as substrate.

### NO levels are higher on solid medium than in liquid medium

**Figure 4:**
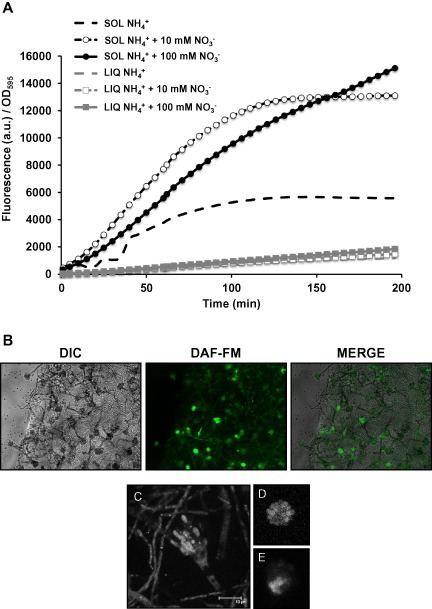
NO production is higher on solid medium than in liquid medium. A. *A* *. nidulans* wild‐type strain was grown in liquid or solid media for 18 h. In liquid medium, *A* *. nidulans* was grown in ammonium liquid medium for 12 h. The mycelia were filtered, washed and transferred to ammonium (LIQ NH_4_^+^), ammonium + 10 mM nitrate (LIQ NH_4_^+^ + 10 mM NO_3_^−^) and ammonium + 100 mM nitrate (LIQ NH_4_^+^ + 100 mM NO_3_^−^). The cultures were grown for 6 additional hours (total growth time was 18 h). On solid media spores were inoculated on solid media containing ammonium (SOL NH_4_^+^), ammonium + 10 mM nitrate (SOL NH_4_^+^ + 10 mM NO_3_^−^) and ammonium + 100 mM nitrate (SOL NH_4_^+^ + 100 mM NO_3_^−^), and the plates were incubated at 37°C for 18 h. DAF‐FM DA was added to all cultures to quantify NO production, and fluorescence was recorded for 200 min. The plot shown is the average of two replicate samples of a representative experiment. B–E. *A* *spergillus* wild‐type strain was grown on solid ammonium medium for 24 h. Agar cubes containing fungal biomass were submerged in a DAF‐FM DA solution for 20 min and washed three times. Conidiophores were imaged by fluorescence microscopy (B) or confocal microscopy (maximal projection in C, and single planes in D–E).

During the previous experiments, we observed a higher production of NO during growth on solid media than in liquid media. In order to compare the NO levels in both type of media, the *A. nidulans* wild‐type strain was grown in liquid or solid media containing ammonium (control) or ammonium supplemented with 10 or 100 mM nitrate for 18 h. DAF‐FM was added to the samples, and fluorescence was recorded for 200 min. Interestingly, production of NO was much higher when *A. nidulans* was grown on solid media than in submerged culture under these experimental conditions (Fig. 4A). Additionally, the increment in the production of NO in the presence of nitrate was also significantly higher on solid media.

In order to find out whether NO was produced or accumulated in hyphae or conidiophores, *Aspergillus* was grown on solid medium. DAF‐FM DA was added and allowed to permeate the cells for 20 min before washing out the excess of the dye. Visualisation under the fluorescent microscope revealed that both hyphae and conidiophores were stained; however, the signal was stronger in the conidiophores than in the hyphae (Fig. 4B). Under this low magnification and using a fluorescent microscope, the signal appeared to be greater in sterigmata cells (metulae and phialides) than in the stalk cells. Confocal microscopy confirmed that the fluorescent signal could be detected in the sterigmata cells (Fig. 4C–E), but also in the vesicle and stalk cells, and in hyphae (Fig. 4C).

### Fungal growth mediates nitrite and nitrate synthesis in ammonium media

**Figure 5:**
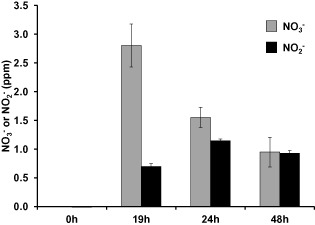
Quantification of nitrate and nitrite in the culture media. *A* *. nidulans Δnia* *D* strain was grown on the surface of a petri dish containing liquid media for the indicated time points. Nitrate (light grey bars) and nitrite (dark grey bars) were quantified with the Griess reagent. Neither nitrate nor nitrite was detected in the media before fungal growth.

The high levels of NO observed on ammonium were intriguing, but the fact that the *ΔniaD* strain produced less NO than the wild type on ammonium solid medium (Fig. 3A) posed two interesting questions. The first one is: can NO be synthesised from nitrate in a medium containing ammonium as sole nitrogen source? This suggests that nitrate could be present in this medium. To test this hypothesis, we employed the *A. nidulans ΔniaD* strain, which does not metabolise nitrate allowing its accumulation, and quantified both nitrate and nitrite during growth in the media. The *ΔniaD* strain was grown on the surface of a petri dish containing liquid media supplemented with ammonium as sole nitrogen source, as previously described (Ruger‐Herreros *et al*., 2011). Under these conditions, *A. nidulans* conidiation proceeded as on solid media. Neither nitrate nor nitrite could be detected in the culture media before the inoculation with the fungal spores. But surprisingly, both nitrate and nitrite were found in the culture media after 19, 24 and 48 h of fungal growth (Fig. 5). When the experiment was performed with a wild‐type strain, nitrate and nitrite were also found in the media but at lower levels, due to the metabolism of nitrate by an active nitrate reductase (Supplementary Fig. S1).

### niaD is induced during conidiation

**Figure 6:**
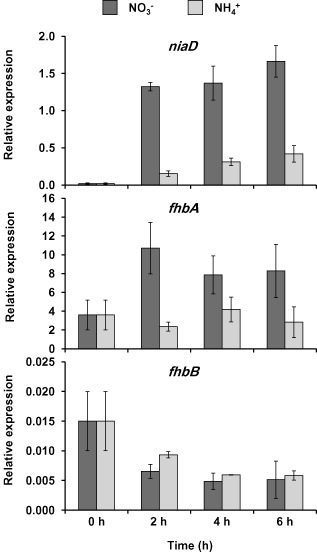
Expression of the flavohaemoglobins and nitrate reductase genes in media containing nitrate or ammonium as sole nitrogen source early during conidiation. *A* *. nidulans* wild‐type strain was grown vegetatively in ammonium liquid minimal medium for 18 h and then transferred to nitrate (dark grey bars) or ammonium (light grey bars) solid medium to induce conidiation. Samples were taken at the indicated time points for RNA isolation. *fhb* *A* *, fhb* *B* and *nia* *D* expression was quantified by real time RT‐PCR. Data were normalised against the expression of the ß‐tubuline gene (*ben* *A*). The plots show the average and standard error of the mean of the relative expression values in at least three independent experiments.

The second question is: if according to the theory of nitrogen regulation, the nitrate reductase gene *niaD* must be repressed in a media containing the repressing nitrogen source ammonium (Arst and Cove, 1973), how is the nitrate reductase active in these conditions? Transcriptomics data obtained previously suggested that the nitrate utilisation pathway was expressed during induction of development on ammonium nitrate medium (Canovas *et al*., 2014). To further confirm this, we first obtained wild‐type vegetative mycelia grown in ammonium liquid medium for 18 h, and then, conidiation was induced by transferring the mycelia to solid media containing ammonium or nitrate as a sole nitrogen source (Fig. 6). Two hours after the induction of conidiation, *niaD* mRNA levels were roughly 70‐fold higher on nitrate solid media than in vegetative mycelia and, strikingly, *niaD* was also transcribed on ammonium solid media. However, the nitrate‐induced level was 8.6‐fold higher compared with ammonium. Longer induction of conidiation on nitrate solid media did not result in much further increase of *niaD* expression (from 70‐ to 92‐fold induction compared with vegetative mycelia). However, longer induction of conidiation on ammonium media increased the induction of *niaD* from 8.5‐ to 23‐fold relative to vegetative mycelia.

We also quantified the levels of the flavohaemoglobin mRNAs after the induction of conidiation. As previously reported, *fhbA* was induced by nitrate (Schinko *et al*., 2010), and there was no significant variation in the expression levels in ammonium during the first hours of conidiation (Fig. 6). On the other hand, the *fhbB* gene was slightly repressed immediately after induction of asexual development regardless of the nitrogen source and dropped threefold after 2 h (Fig. 6). Downregulation of *fhbB* was similar in both nitrogen sources ammonium and nitrate, suggesting that *fhbB* is not constitutive, but rather developmentally regulated.

### NO levels are increased during the first hours of conidiation

**Figure 7:**
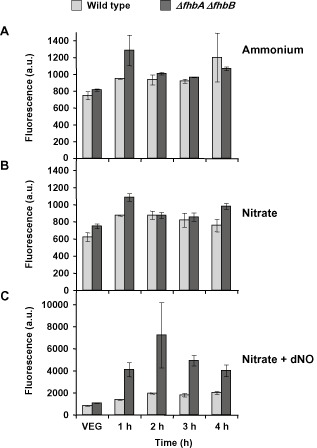
NO quantification after induction of development. *A* *. nidulans* wild‐type strain (light grey bars) and a *Δfhb* *A* *Δfhb* *B* mutant (dark grey bars) were grown vegetatively in ammonium liquid medium for 18 h (VEG) and then transferred to solid media containing different nitrogen sources to induce development: ammonium tartrate as control nitrogen source (A), sodium nitrate (B) and sodium nitrate supplemented with NO‐releasing chemical compound dNO (C). Samples were taken at different times, and the fluorescence produced by the reaction of NO and DAF‐FM DA was quantified. Please note the different graph scales in A–C.

The higher levels of NO produced during growth on solid media compared with liquid media suggests that there might be a connection between NO and development. Indeed the expression pattern of *fhbB* points to an early regulation of the NO levels during the transition from vegetative growth to conidiation. In order to study this possible connection between NO and asexual development, we first quantified the NO levels at the early stages of the transition from vegetative growth to conidiation. The wild‐type strain and the mutant lacking both flavohaemoglobins (*ΔfhbA ΔfhbB*), which cannot detoxify NO, were grown in ammonium liquid medium, and then transferred to solid media to induce conidiation as above. Samples were taken at 1 h intervals, and NO was quantified with DAF‐FM. As can be seen in Fig. 7A–B, NO increased after 1 h of induction compared with vegetative growth in all the conditions. In fact, we observed this increase after induction of conidiation for 1 h, and it remained elevated at least for 7 h (data not shown). There were no major differences in the NO levels between ammonium and nitrate growth conditions during the early induction of conidiation.

We were interested in creating a higher range of NO levels for the following experiments. Therefore, we added the NO‐releasing chemical compound detaNONOate (dNO), which was previously tested for increased levels of NO (Fig. 1E), and checked for an additional increase of NO levels (Fig. 7C). In this case, we found a drastic increase of NO in both strains, as expected, and NO levels were threefold higher in the mutant than in the wild type.

### Conidiation (asexual development) is repressed by elevated NO levels

**Figure 8:**
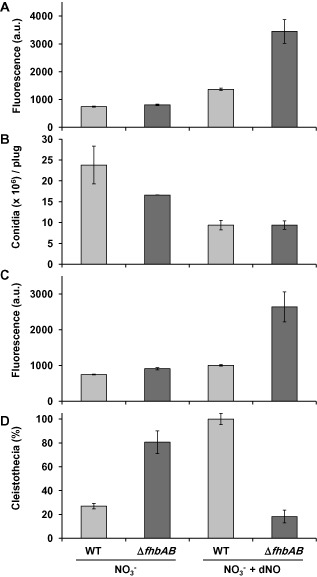
Effects of NO on asexual (conidiation) and sexual reproduction. A and B. Wild type and *Δfhb* *A* *Δfhb* *B* strains were grown in minimal media for 18 h and then transferred to nitrate solid medium with or without 1.5 mM dNO. The plates were incubated at 37°C for 1 h for NO quantifications (**A**) or for 72 h for conidial counting (**B**). Data show the average of at least three independent experiments and standard error of the mean. C and D. Wild type and *Δfhb* *A* *Δfhb* *B* strains were grown in minimal media for 18 h and then transferred to nitrate solid medium with or without 1.5 mM dNO. The plates were sealed and incubated at 37°C in the dark for 1 h for NO quantifications (**C**) or for 15 days for the quantification of the number of cleistothecia. Data are given as the percentage of WT grown in nitrate + dNO (**D**). Data show the average of at least three independent experiments and standard error of the mean.

In order to get insight into the effects of NO during reproduction, we first tested the effects of different NO levels on conidiation using the wild‐type strain and the mutant lacking both flavohaemoglobins (*ΔfhbA ΔfhbB*). Conidiation was induced as described above by transferring vegetative mycelia of both strains to solid media with or without dNO. Based on the results shown above, we expected that these conditions and strains generated a gradient of NO. As NO levels quickly increased after induction of conidiation, we quantified the levels under these conditions and observed the expected gradient of increasing NO levels (Fig. 8A). After 3 days of incubation, the number of conidia was counted. Conidiation was slightly reduced in the double mutant compared with the wild type in the control nitrate media (Fig. 8B). Addition of the NO‐donor dNO to the media resulted in a drastic increase of NO levels, especially in the mutant strain, and a further reduction of conidiation (ca. 60%) both in the wild type (*P* < 0.1; Student's *t*‐test) and in the mutant strain (*P* < 0.05; Student's *t*‐test). Observation of the fungal growth on the solid media with a stereoscopic microscope revealed a reduction in the density of conidiophores (Fig. S2). These results suggest that an increase of NO correlates with a decrease in conidiation.

### Sexual development is induced by NO

Conidiation and sexual development are normally balanced in *A. nidulans*, meaning that when one of them increases, the other one decreases (Adams *et al*., 1998; Rodriguez‐Romero *et al*., 2010; Dyer and O'Gorman, 2012). To study the effects of NO in sexual development of *A. nidulans*, we followed a similar strategy as described above but growing strains under conditions to induce sexual development (i.e., sealed plates in the dark). Again, this strategy also allowed creating the NO gradient under the conditions to induce sexual development (Fig. 8C). Opposite to the effects observed in conidiation, there was an increase in the number of cleistothecia (the sexual reproductive structures of *A. nidulans*) in the mutant strain compared with the wild type in the control medium (Fig. 8D). When the NO‐releasing compound dNO was added to the media, the number of cleistothecia even increased in the parental strain compared with the control media (Fig. 8D). Similar results were obtained by Baidya *et al*. (2011). This increase in the number of cleistothecia correlated with the increase of NO. However, addition of dNO to the double *ΔfhbAΔfhbB* mutant resulted in the lowest number of cleistothecia, which was the opposite to what could be expected based on the other samples. We reasoned that this may be due to toxicity from the excessive amounts of NO in our experimental set up, which were more than twofold compared with the amounts of NO produced by the wild‐type under the same conditions (Fig. 8C). Baidya and collaborators found different results in their experiments using the strains and conditions developed in our laboratories: the number of cleistothecia was not reduced upon addition of dNO to the flavohaemoglobins mutant. However, they did not quantify the NO levels of the corresponding samples (Baidya *et al*., 2011).

In order to monitor the toxic effect of the NO radicals, we quantified the radial growth rate on solid nitrate media of the wild type and the double mutant (Supplementary Fig. S3). Addition of dNO resulted in a slight decrease of the wild‐type growth rate and in a drastic decrease of the mutant (56% of the wild‐type growth rate under the same conditions). This suggests that dNO was indeed exerting a toxic effect under our experimental conditions, particularly in the case of the flavohaemoglobin mutant, which lacks the NO detoxification enzymes.

### Different NO levels affect the expression of the regulator of sexual development nsdD and the regulator of conidiation brlA

**Figure 9:**
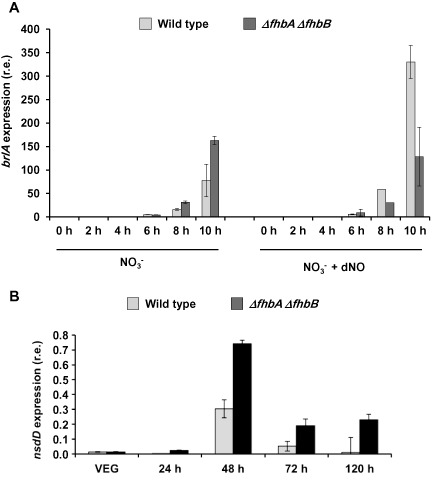
Expression of developmental regulators during asexual or sexual reproduction at different NO levels. A. *brl* *A* expression during conidiation. *A* *. nidulans* wild type and *Δfhb* *A* *Δfhb* *B* strains were grown in liquid minimal medium for 18 h and then transferred to nitrate solid medium with or without dNO to induce conidiation. B. *nsd* *D* expression during sexual development. *A* *. nidulans* wild type and *Δfhb* *A* *Δfhb* *B* strains were grown in liquid minimal medium for 18 h and then transferred to nitrate solid medium with or without dNO. Plates were sealed and incubated in the dark to induce sexual development. Samples were taken at the indicated time points for RNA isolation. *brl* *A* and *nsd* *D* expression was quantified by real‐time RT‐PCR. Data were normalised against the expression of the ß‐tubuline gene (*ben* *A*). The plot shows the average and standard error of the mean of the relative expression values in at least three independent experiments.

Using the same conditions as above, the expression of the master regulator of conidiation *brlA* was quantified at different time points after induction of conidiation (Fig. 9A). The expression pattern of *brlA* was similar in all the conditions tested, i.e. we did not observe that the expression levels of *brlA* correlated with the number of conidia. In fact, the highest value of *brlA* expression was found at 10 h in the wild type grown with dNO, which corresponds to one of the conditions with the lowest number of conidia. This suggests that the effects of NO on conidiation were not mediated through the control of the *brlA* expression levels.

To further study the correlation between NO levels and number of cleistothecia, (Fig. 8C–D) sexual development was induced in the wild‐type strain, and the accumulation of mRNA of *nsdD* was quantified. NsdD is a GATA‐transcription factor necessary to activate sexual development (Han *et al*., 2001). As shown in Fig. 9B, *nsdD* mRNA accumulation increased up to 48 h and then decreased. *nsdD* expression was higher in the flavohaemoglobin mutant than in the wild‐type strain in all the time points tested, and it still remained relatively high until 120 h, suggesting that NO could play a role in the induction of the transcription of *nsdD*. Interestingly, opposite to the conidiation regulator *brlA*, the effect of NO in the increased expression of the sexual developmental regulator *nsdD* correlated with the amount of cleistothecia. However, whether the modulation of expression of these regulators by NO is direct or indirect still remains to be addressed.

### fhbA and fhbB are regulated differently during conidiation

**Figure 10:**
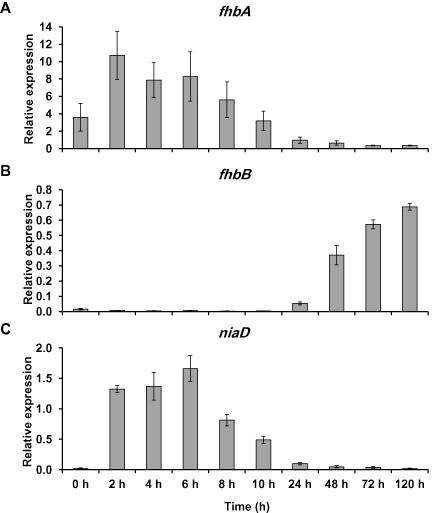
Expression of the flavohaemoglobin and nitrate reductase genes during conidiation. *A* *. nidulans* wild‐type strain was grown in ammonium liquid minimal medium for 18 h and then transferred to nitrate solid medium to induce conidiation. Samples were taken at the indicated time points for RNA isolation. *fhb* *A* (A), *fhb* *B* (B) and *nia* *D* (C) expression was quantified by real‐time RT‐PCR. Data were normalised against the expression of the ß‐tubuline gene (*ben* *A*) and it is shown as relative to vegetative growth. The plots show the average and standard error of the mean of the relative expression values in at least three independent experiments.

As the NO levels were regulated during conidiation, we studied the expression of the NO‐metabolising flavohaemoglobin genes *fhbA* and *fhbB* for a longer period after induction of conidiation. The wild‐type strain was grown in ammonium liquid medium and then transferred to nitrate solid medium. Samples were again taken at different hours after induction of conidiation, and the mRNA levels of both genes *fhbA* and *fhbB* were quantified (Fig. 10). Both flavohaemoglobins were expressed during conidiation, but they showed distinct patterns of expression. In particular, *fhbA* expression was induced in the first hours and then gradually decreased (Fig. 10A), whereas *fhbB* expression was low during the first hours, and then was induced from 24 h until 120 h (Fig. 10B). These results suggest that both flavohaemoglobins are regulated differently during NO metabolism, which may reflect different biological roles in the control of the homeostasis of the regulatory molecule NO.

Furthermore, it was also interesting to find that the expression of *niaD* was induced during the early stages reaching a maximum at 6 h after the transition to conidiation (91‐fold induction with respect to vegetative growth) and then it started to decrease, showing an expression pattern very similar to the nitrogen regulated *fhbA* gene (Fig. 10C).

### areA and nirA regulate the expression of niaD and fhbA during conidiation

**Figure 11:**
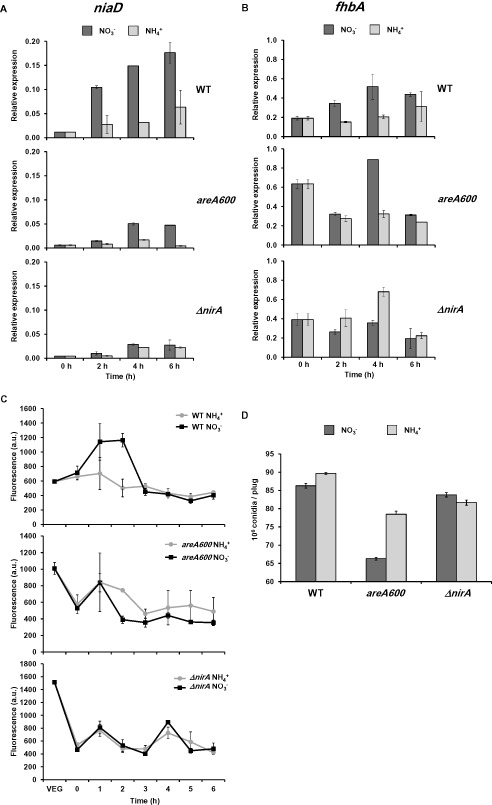
Induction of *nia* *D* and *fhb* *A* during conidiation requires functional *are* *A* and *nir* *A*. A and B. Conditions are the same as stated in Fig. 5. Wild type, *are* *A* *600* and *Δnir* *A* strains were transferred to nitrate (dark grey bars) or ammonium (light grey bars) solid media to induce conidiation. The expression of *nia* *D* (**A**) and *fhb* *A* (**B**) was quantified by real‐time RT‐PCR. C. NO levels were quantified as in Fig. 6 in the wild‐type strain and *are* *A* *600* and *Δnir* *A* mutants on nitrate (dark grey squares) or ammonium (light grey circles). VEG indicates samples that were always maintained in submerged cultures, and time 0 indicates samples that were employed to quantify NO levels immediately after transferring to a solid surface. D. Conidia were counted in the three strains 72 h after the transfer to a solid surface. In all cases, data shown is the average of three independent samples and the standard error of the mean.

We questioned whether the general regulator of nitrogen metabolism, *areA*, and the nitrate pathway specific regulator, *nirA*, were required for the induction of *niaD* and *fhbA* during the induction of conidiation. Wild‐type, *areA600* and *ΔnirA*, strains were grown in ammonium liquid media and then transferred to nitrate or ammonium solid media. In the *areA*600 loss‐of‐function mutant, the *niaD* mRNA levels were three‐ to fourfold lower than in the wild type after induction of conidiation on nitrate (Fig. 11A). Expression on ammonium was also reduced, particularly at 6 h. However, in both media induction was not completely abolished suggesting that other factors might also contribute to the induction. Deletion of the pathway specific regulator *nirA* also resulted in a strong decrease of the expression levels in both media.

We found an increased expression of *fhbA* during vegetative growth in both mutants (Fig. 11B). This result, although it might be surprising, was also reported by Schinko *et al*. (2010), and it might result from an *areA* and *nirA*‐mediated repression on ammonium. In both *areA600* and *ΔnirA* mutants, there was no induction of *fhbA* after the transfer to solid media, suggesting that under these experimental conditions both regulators are required for induction of *fhbA*.

The opposing activities (synthesis and metabolism of NO) were regulated in a similar way in the wild type, which made us wonder whether this would result in higher or lower levels of NO. As expected, the wild‐type strain showed an immediate increase in the NO levels upon transfer from liquid to solid nitrate media. Unlike the wild‐type strain, neither the *areA* nor the *nirA* mutants were capable of producing increased NO levels upon transfer to solid media regardless of the nitrogen source (Fig. 11C). Significantly, the NO levels were higher during vegetative growth in liquid media in the mutants, which may lead to greater levels of *fhbA* expression (through an unknown mechanism). Surprisingly, this reduction of the NO levels was not accompanied by an increase in the conidiation levels, but rather by a slight reduction of 9–13% on ammonium (Fig. 11D). The difference was higher on nitrate media in the case of the *areA* mutant (23% decrease).

## Discussion

While the pathways for the synthesis of NO are clear and well studied in mammals and plants (Alderton *et al*., 2001; Gorren and Mayer, 2007; Gupta *et al*., 2011), in fungi, they have remained elusive so far. A recent and very thorough study in *Magnaporthe* could not provide any evidence of the putative routes and genes for the biosynthesis of NO (Samalova *et al*., 2013). In this work, we provide evidence for the existence of a nitrate‐reductase dependent route involved in the biosynthesis of NO in *A. nidulans*. Although this route is functional during growth in both liquid and solid media, it is more active on solid media where reproductive development occurs.

The expression pattern of *niaD* and *fhbA* after induction of development in nitrate media is similar, probably as a consequence of the nitrate‐dependent regulation of both genes that required functional *areA* and *nirA* genes. However, they showed a different pattern in ammonium media, where *niaD* but not *fhbA* was induced. These differences in the regulation between both genes allow the fungus to produce similar NO levels in both media during the induction of conidiation. Importantly, we have shown that the *niaD* route is operative during conidiation even in the presence of the repressing nitrogen source ammonium. It is well known from studies in liquid media that nitrogen metabolite repression (e.g. in the presence of ammonium) leads to loss of *niaD* expression. This regulation is mediated by inactivation of the GATA‐type zinc finger protein AreA, which normally acts as co‐activator for many genes, under nitrogen metabolite repression (Arst and Cove, 1973; Caddick *et al*., 1986; Burger *et al*., 1991; Marzluf, 1997; Todd *et al*., 2005; Berger *et al*., 2006). The question is how the ammonium‐repression is overcome to induce *niaD*. For example, the glucose‐repressed *acuD* gene, which is involved in the utilisation of alternative two‐carbon molecules (such as acetate or ethanol), is regulated during development by the conidiation regulators BrlA and AbaA in *Penicillium marneffei* (Canovas and Andrianopoulos, 2006). However, no consensus sequences for the binding sites of either AbaA or BrlA could be found in the promoter region of *niaD*. Actually, *niaD* gene expression during conidiation required functional *areA* and *nirA* genes. Induction of *fhbA* was also abolished in both mutants, although *fhbA* was reported to be regulated only by *nirA*, and not by *areA* (Schinko *et al*., 2010). However, there are four AreA consensus binding sites in the promoter of *fhbA*, which points to an AreA‐dependent regulation of *fhbA* only during induction of conidiation and not during vegetative growth. This unique mechanism would require additional players to act selectively during this specific stage of the fungal life cycle (for example, six GATA sites were found in that region) or specific post‐transcriptional modifications of AreA, which allow it to override the repression imposed by the presence of ammonium in the media to induce *niaD* expression in the early stages of conidiation. As the expression levels of *areA* are rather constant during the entire life cycle, post‐transcriptional modifications are the most feasible explanation. It could be argued whether this induction requires developmental or endogenous signals. In principle to propose an endogenous induction (i.e. by intracellular accumulation of nitrate), in addition to de‐repression, expression of *niaD* would also require nitrate or nitrite. Indeed, when *A. nidulans* was grown in ammonium media, both nitrate and nitrite were found in the media after culturing the fungus. Nitrate could originate from the oxidation of NO (Schinko *et al*., 2013). An alternative option would involve heterotrophic nitrification pathways. This process has not been studied in detail, and we could only find two reports in the literature demonstrating the formation of nitrate and nitrite through unknown mechanisms by *Aspergillus flavus* in media containing ammonium in a pH‐dependent manner (Hirsch *et al*., 1961) or in media containing certain amino acids (Hatcher and Schmidt, 1971). The data presented here suggest that a considerable amount of NO produced in ammonium media is *niaD*‐dependent and, therefore, it is produced through the nitrate route. As not all the NO was produced by the nitrate reduction pathway, it implies that there must be additional routes for the synthesis of NO in fungi. The most plausible alternative would be from arginine by an alleged nitric oxide synthase. However, work published in *Magnaporthe* could not prove this hypothesis, despite knocking out all putative genes (Samalova *et al*., 2013) and consequently the additional routes and sources of NO still remain unknown in fungi.

Although, first, it puzzled us to observe that the NO levels were lower when the strains were grown in nitrate compared with ammonium, the nitrate‐dependent induction of *fhbA* can explain this reduction. Indeed, addition of nitrate to repressing ammonium or non‐repressing proline media only provoked modest increases in the NO levels. *fhbA* is expressed in nitrate media even in the presence of repressing nitrogen sources, such as ammonium (Schinko *et al*., 2010). Consequently, deletion of *fhbA* allowed drastic increases of the NO levels upon addition of nitrate to the media. Other authors have observed that both *ΔfhbA* and *ΔfhbA ΔfhbB* mutants display the same phenotype with a promoting effect of sexual development, but the *ΔfhbB* mutant does not (Baidya *et al*., 2011). This agrees with our data that *fhbB* but not *fhbA* is developmentally repressed upon induction of development, and consequently, deletion of *fhbA* would allow a higher increase of the NO levels at the beginning of development. On the other hand, deletion of *fhbB* cannot have a major impact in the early decisions, as this gene is repressed immediately after induction of conidiation, but rather in steady state levels of NO as it was expressed 24 h after induction of conidiation. The two fungal flavohaemoglobins are localised in different subcellular compartments (te Biesebeke *et al*., 2010), which also support the idea of playing different biological roles during the life cycle of the fungus. Collectively, the levels of NO could be explained by a co‐ordinated action of the flavohaemoglobins during development, acting first the cytoplasmic *fhbA*, and then the mitochondrial *fhbB* in late development. The role of the flavohaemoglobins in NO metabolism was previously reported in *A. nidulans* (Schinko *et al*., 2010), and here we further confirmed with additional new data their role in the fine tuning of the NO metabolism and concentration during development. Another factor that might influence an increase levels of NO during sexual development over conidiation is the lowering of oxygen availability, which would decrease the rate of NO oxidation (i.e. conversion back to nitrate), both spontaneous and/or mediated by the haem‐containing flavohaemoglobins. Two additional genes have recently been implicated in the response to nitrosative stress in *A. nidulans*. The porphobilinogen deaminase *hemC* acts by promoting the activity of the flavohaemoglobins through an unknown mechanism, whereas the NO‐inducible nitrosothionein *ntpA* scavenges NO through S‐nitrosylation (Zhou *et al*., 2012; 2013). However, the possible role of these two genes during conidiation and sexual reproduction still remains to be studied.

At 24 h, the mitochondrial flavohaemoglobin *fhbB* expression is induced threefold and at 48 h this induction boosts over 20‐fold, suggesting that it might contribute to control the NO levels. This time coincides with the maximal expression of the sexual regulator *nsdD* and could contribute to the balance between conidiation and sexual development. Indeed, dissimilatory nitrate reduction, in which nitrate is employed as a terminal acceptor of electrons, and production of NO have been found in *Aspergillus terreus* under anoxic conditions (Stief *et al*., 2014). This dissimilatory reduction of nitrate producing ammonium was found to be cytosolic, and involves the assimilatory nitrate and nitrite reductases (*niaD* and *niiA*) coupled to ethanol oxidation under anoxic conditions in *A. nidulans* (Takasaki *et al*., 2004). Taken altogether, it suggests that the assimilatory NR is responsible for the synthesis of NO working as a dissimilatory NR under anoxic conditions. If this hypothesis is true, the dissimilatory nitrate reductase activity must be restricted to a secluded area of the mycelial mat in close contact with the solid surface, but isolated from the air interface.

The levels of the signalling molecule NO also increased immediately after the induction of conidiation suggesting that it could be one of the earliest signals appearing during the transition from vegetative mycelia to the reproduction modes. This increase in the NO levels could be explained with the repression of the expression of the flavohaemoglobin *fhbB* and the induction of the nitrate reductase *niaD*. The increase of NO seemed to favour the sexual reproduction program while reducing the conidiation one. In the case of the sexual reproductive mode, increasing the levels of NO provoked the increased expression of the sexual regulator *nsdD* and the number of cleistothecial structures. Such correlation was not found in the case of the number of conidia and *brlA* expression. However, mutation of *areA* or *nirA* abolished the induction of *niaD* expression and the subsequent increased in NO levels. Blocking this increase of NO levels did not result in the expected increase in conidiation. One hypothesis that agrees with these data is that the induction of the reproduction programmes under laboratory conditions is a several‐step process, in which the first step is the commitment for the transition between a vegetative growth style to the reproduction program, and then the fungus regulates the balance between asexual and sexual programmes depending on the environmental factors. Considering the greater signal observed in conidiophores by fluorescence microscopy, NO could play different roles at the different stages.

In conclusion, here we report that the nitrate reductase is involved in the synthesis of NO from nitrite in *Aspergillus*. *niaD* and *fhbA* are expressed even in the presence of the repressing nitrogen source ammonium during conidiation in a NirA‐ and AreA‐dependent manner, which contributes together with a temporal repression of *fhbB* to a transient increase in the NO levels upon induction of conidiation.

## Experimental procedures

### Strains, media and culture conditions

Strains used in this study are listed in Table S1. Strains were grown in complete or minimal media containing the appropriate supplements at 37°C (Cove, 1966). One per cent glucose was used as carbon source. Ammonium, nitrate, nitrite and/or proline were used as nitrogen sources as indicated. The NO chemical donor 3,3‐bis(aminoethyl)‐1‐hydroxy‐2‐oxo‐1‐triazene, also called detaNONOate (dNO) and the NO‐scavenger 4,4,5,5‐tetramethylimidazoline‐l‐oxyl3‐oxide (PTIO) were purchased from Sigma.

### RNA isolation and real‐time RT‐PCR

Isolation of RNA and quantification of mRNA was performed as previously described (Ruger‐Herreros *et al*., 2011). Briefly, *Aspergillus* mycelia (100–200 mg) were disrupted in 1 ml of TRI reagent (Sigma) with 1.5 g of zirconium beads using a cell homogeniser (FastPrep‐24, MP Biomedicals). Cell debris was removed by centrifugation. Supernatants were extracted with chloroform, and RNA was precipitated with isopropanol. RNA samples were further purified using the NucleoSpin RNA II Nucleic Acid and Protein Purification Kit (Macherey‐Nagel).

The primers employed for real‐time RT‐PCR are detailed in Table S2. Real‐time RT‐PCR experiments were performed in a LightCycler 480 II (Roche) using the One Step SYBR® PrimeScript™ RT‐PCR Kit (Takara Bio). The fluorescent signal obtained for each gene was normalised to the corresponding fluorescent signal obtained with the ß‐tubulin gene *benA* to correct for sampling errors. Expression data are the average of at least three independent replicates.

### Developmental biology experiments

Strains were grown in liquid medium for 18 h at 37°C and then transferred to solid media. Plates were incubated under light for conidiation conditions, or alternatively they were sealed and incubated in the dark for sexual developmental conditions. Plugs were cut out from the plate 72 h after induction of development. Conidia in the plugs were resuspended in Tween 0.1% buffer and counted. For counting cleistothecia, plates incubated for 15 days were first sprayed with 70% ethanol to facilitate the visualisation of the sexual structures and then photographed. Data shown are the average of at least four independent experiments.

### NO quantification

Nitric oxide was quantified in samples by using the NO‐sensitive fluorescent dye DAF‐FM DA (Invitrogen) following the manufacturer's instructions except in Fig. 1, in which DAF‐FM (Sigma) was also employed. In Fig. 1, fungal cells were grown in liquid ammonium minimal medium for 16 h, and 50 μl of 5 μM DAF‐FM DA or DAF‐FM were added to 100 μl of each sample. Cells were loaded with the dye for 20 min and washed three times with a 0.05% tween solution (when indicated). Samples were transferred to 96‐well plates, and fluorescence was recorded in a Synergy HT Multi‐mode Microplate Reader (Biotek) equipped with FITC filter sets (**λ_Em_** 488 nm, **λ_Ex_** 520 nm). In Fig. 2A and B, conidia were inoculated into 100 μl of minimal media at a final concentration of 10^5^ conidia ml^−1^ in 96‐well plates, and the plates were incubated with shaking at 37°C for 16 h to allow aeration of the culture. After incubation, DAF‐FM DA was added to the cultures to a final concentration of 2.5 μM, and fluorescence was recorded in POLARstar Omega fluorometer (MG Labtech) equipped with FITC filter sets (**λ_Em_** 488 nm, **λ_Ex_** 520 nm) for up to 200 min. Data were analysed with Omega Control (version 1.10) software. For the rest of the experiments, the strains were grown in ammonium liquid medium for 12 h, then transferred to liquid media containing the appropriate nitrogen sources and incubation continued for 6 additional hours. Cells were loaded with the dye for 20 min and transferred to 96‐well plates. Alternatively, the strains were grown in ammonium liquid medium for 18 h and then transferred to solid media for the indicated time points. Plates were incubated under the conditions described above for conidiation or sexual development. Samples were taken from the plates at the indicated time points, submerged in a solution of 5 μM DAF‐FM DA for 1 h. Samples were centrifuged, and the supernatant was transferred to a 96‐well plate for fluorescence quantification. For the other experiments performed on solid media in Figs 3 and 4, 10^5^ conidia were inoculated on 96‐well plates containing solid media containing the indicated nitrogen sources and incubated for 18 h before quantification of NO. In Fig. 3B, fungal cells were grown in proline solid media for 18 h, fluorescence quantification started after addition of DAF‐FM DA and then the indicated nitrogen sources were added and fluorescence quantification continued for 2 additional hours. In all cases 2.5 μM DAF‐FM DA was added to the samples. Fluorescence was detected and quantified in a Synergy HT Multi‐mode Microplate Reader (Biotek) equipped with GFP filter sets. Data were analysed with Gen5^TM^ Data Analysis Software. In all experiments, data were normalised to dry weight or OD_595_ as indicated. Experiments were repeated at least two times and performed in duplicates or triplicates, depending on the experiment.

### Microscopy

*Aspergillus* was grown either on liquid ammonium medium for 14–16 h or solid ammonium medium for 24 h. A solution of 5 μM DAF‐FM DA (solid and liquid samples) or DAF‐FM (liquid samples) was added to the mycelium. After cell loading of the dye for 20 min, the excess of dye was washed out with a 0.05% Tween 80 solution. Images were recorded in an Olympus IX2‐UCB inverted fluorescence microscope equipped with a FITC filter set (λ_Em_ 488 nm; λ_Ex_ 520 nm). Images in Fig. 1C were taken using identical settings for comparative purposes. Images in Fig. 4B were processed and merged using Adobe ImageReady CS2 (Adobe Systems Incorporated, CA, USA). Confocal images were obtained in a ZEISS LSM 7 DUO confocal microscope with an excitation at 488 nm using an OPSS laser beam. Confocal images were processed using ZEN Lite 2012 (Zeiss, Germany).

### Nitrate and nitrite measurements in the culture supernatant

Nitrate and nitrite were quantified in the culture media using the Griess reagent as previously described (Schinko *et al*., 2010).

## Supporting information

Supporting information
