# Supplementary material for: Nitric oxide synthesis by nitrate reductase is regulated during development in A spergillus
Source: Mol Microbiol. 2015 Oct 14;99(1):15–33. doi: 10.1111/mmi.13211 (PMC4982101; doi:10.1111/mmi.13211)
Supplement: Supplementary file 1 — Supporting information [file MMI-99-15-s001.zip › MMI_13211_supp-0002-Marcos_et_al_SUP-MAT.pdf]

SUPPLEMENTARY MATERIAL

Table S1

Strains used in this study

Strains	Genotype[1]	Reference
Cib08	*biA1 yA2*	(Schinko *et al*., 2010)
CibAB	*biA1 yA2 ΔfhbA::argB ΔfhbB::argB*	(Schinko *et al*., 2010)
DKA187	*ΔniaD::argB biA1*	(Schinko *et al*., 2010)
DKA119	*ΔniaD::argB ΔfhbA::argB ΔfhbB::argB biA1*	This study
DKA237	*niiA4 ΔfhbA::argB ΔfhbB::argB biA1*	This study
MH11058	*biA1 niiA4 pyroA4 nkuA::bar*	From Michael Hynes
areA600	*areA600 pantoB100 biA1*	(Kudla *et al*., 1990)
TS005	*ΔnirA::AfriboB riboB2 pyroA4 argB2 pyrG89 ΔnkuA::argB*	(Schinko *et al*., 2010)

[1] All strains are *veA1*

Table S2

Primers used in this study.

**Primer name**	**Sequence**
fhbA RT-for	CGCGCCGCTCACAAC
fhbA RT-rev	CAACGAACTTGGCGTTCATG
fhbB RT-for	GCGCATTGGCCATAAACAT
fhbB RT-rev	TCGCCGACGATCGGATAG
niaD RT-for	TCGCTGGATAAGGGCAAATC
niaD RT-rev	CGGTATTTGTCTTCGGCGTATT
brlA-F	TACCGCGACGGGTTTCAG
brlA-R	GAGGTCTGTCGTCGGAGCAT
nsdD RT-for	CAATGCATGCGGCTTACACT
nsdD RT-rev	CCTGCCGCCTGTTTGG
benA-F	CCAGTGTGGTAACCAGGTTGGT
benA-R	GGCGTCGAGGCCATGTT

Supplementary Fig. 1

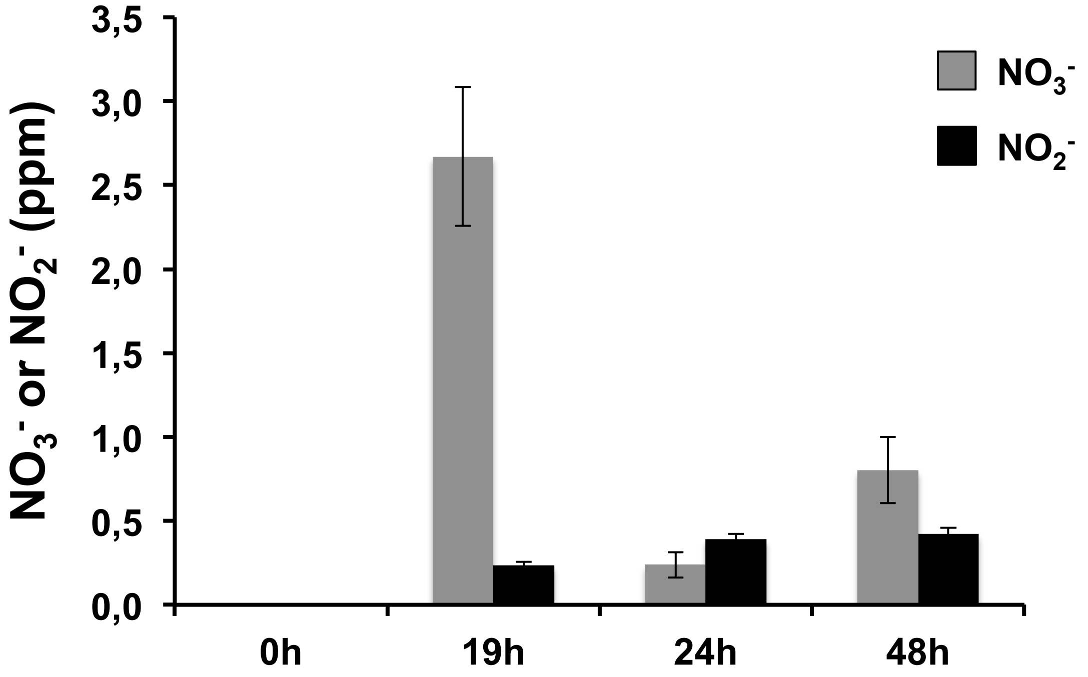

Fig. S1. Quantification of nitrate and nitrite in the culture media.
A. nidulans wiild type strain was grown on the surface of a petri dish containing liquid media for the indicated time points. Nitrate (light grey bars) and nitrite (dark grey bars) were quantified with the Griess reagent. Neither nitrate or nitrite were detected in the media before fungal growth.

Supplementary Fig. 2

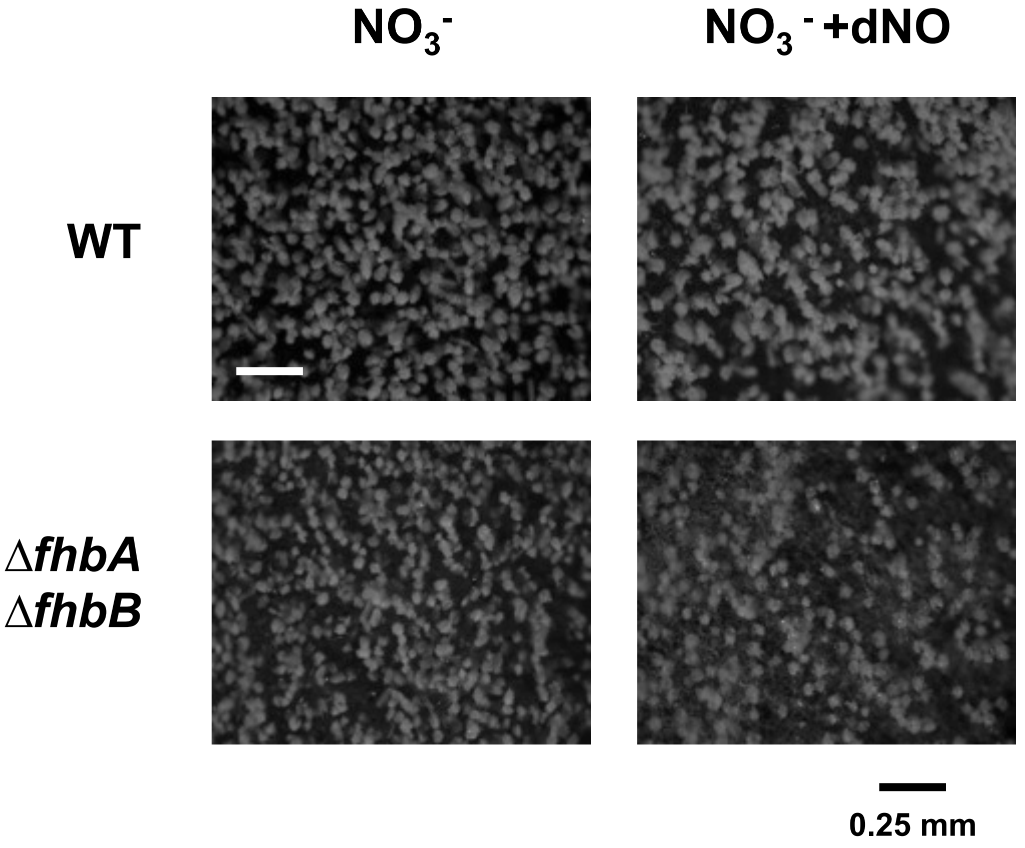

Fig. S2. Addition of the NO-releasing compound detaNONOATE reduces de density of conidiophores.
Stereoscopic microscope images of the wild type and the flavohaemoglobin mutant (Δ*fhbA* Δ*fhbB*) grown on nitrate in the presence or in the absence of dNO for 72 h. A reduction of the conidiophore density was observed in the presence of dNO in both strains. The effect of dNO was more pronounced in the flavohaemoglobin mutant.

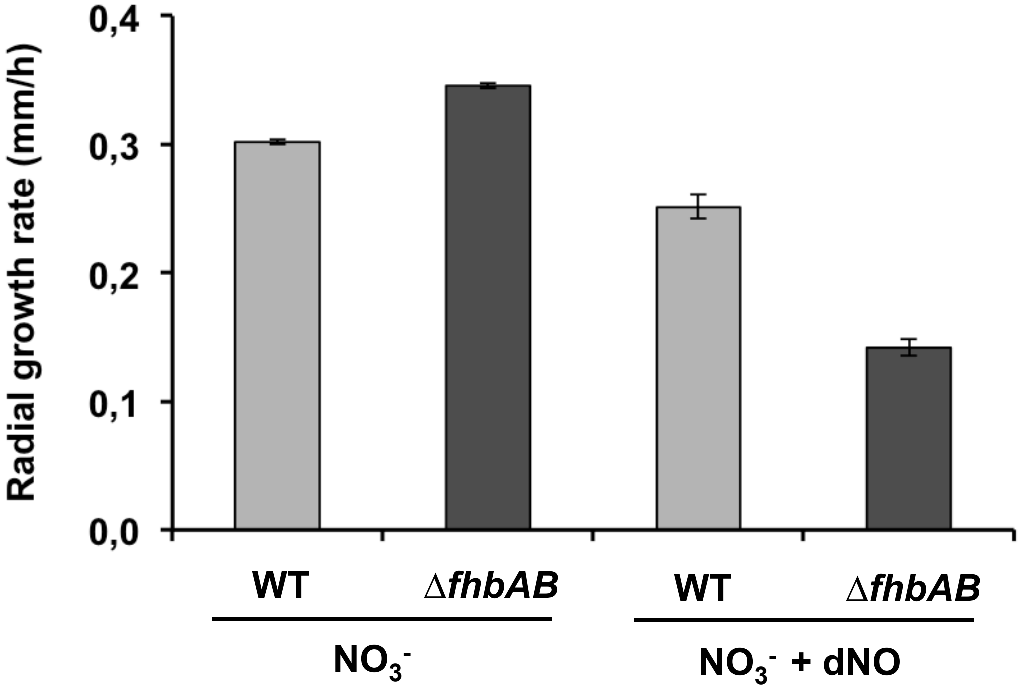

Fig. S3. Flavohaemoglobins are involved in the detoxification of NO.
A. nidulans wild type and Δ*fhbA* Δ*fhbB* strains were grown in minimal media supplemented with nitrate as sole nitrogen source. Nitrate + dNO indicates that 1.5 mM dNO was added to the cultures to further increase the amount of NO. Radial growth was monitored up to 7 days measuring the colony diameter. To calculate the growth rate, we employ a regression line between 2 and 7 days to avoid effects during germination. Data are the average of two independent experiments. Vertical bars indicate standard error of the mean.
